# Why Community Health Systems Have Not Flourished in High Income Countries: What the Australian Experience Tells Us

**DOI:** 10.34172/ijhpm.2021.42

**Published:** 2021-05-18

**Authors:** Fran Baum, Toby Freeman

**Affiliations:** Southgate Institute for Health, Society and Equity, Flinders University, Adelaide, SA, Australia.

**Keywords:** Primary Healthcare, Health Policy, Social Determinants of Health, Community Control, Aboriginal Health

## Abstract

**Background:** Despite the value of community health systems, they have not flourished in high income countries and there are no system-wide examples in high income countries where community health is regarded as the mainstream model. Those that do exist in Australia, Canada, the United States and the United Kingdom provide examples of comprehensive primary healthcare (PHC) but are marginal to bio-medical primary medical care. The aim of this paper is to examine the factors that account for the absence of strong community health systems in high income countries, using Australia as an example.

**Methods:** Data are drawn from two Australian PHC studies led by the authors. One examined seven case studies of community health services over a five-year period which saw considerable health system change. The second examined regional PHC organisations. We conducted new analysis using the ‘three I’s’ framework (interests, institutions, ideas) to examine why community health systems have not flourished in high-income countries.

**Results:** The elements of the community health services that provide insights on how they could become the basis of an effective community health system are: a focus on equity and accessibility, effective community participation/control; multidisciplinary teamwork; and strategies from care to health promotion. Key barriers identified were: when general practitioners (GPs) were seen to lead rather than be part of a team; funding models that encourage curative services rather than disease prevention and health promotion; and professional and medical dominance so that community voices are drowned out.

**Conclusion:** Our study of the community health system in Australia indicates that instituting such a system in high income countries will require systematic ideological, political and institutional change to shift the overarching government policy environment, and health sector policies and practices towards a social model of health which allows community control, and multidisciplinary service provision.

## Introduction

**Key Messages**

** Implications for policy makers**

- Public policy based on neo-liberal principles is a threat to the collectivist and redistributive aims of community health systems.
- Funding models need to be flexible in order to respond to community perspectives and enable health workers and planners to conduct important work that is not easy to measure with short term output criteria.
- A health system is more likely to be based on community health if it accepts and promotes a social health view which enables advocacy and action on social determinants of health.
- Multidisciplinary healthcare requires strong support from health systems and in initial training to be effectively implemented.
- Medical practitioners are a vital part of community health systems, but they should be regarded as a member of teams and not necessarily be the leader or the dominant voice in the system.

** Implications for the public**

Communities in high income countries would receive more integrated care and see more disease prevented and health and well-being promoted if their health systems were based on publicly funded and community-controlled community health systems. Such systems would create a comprehensive model of primary healthcare (PHC) and provide users with a range of services including nursing, medical, physiotherapy, social work and psychology from professionals working in integrated and co-ordinated teams. Social determinants of health are a major influence on population health and so governments should design a health system which is able to take into account their influence on the health of patients and also advocate to other sectors (eg, housing and employment) so they take their impact on health into account and take measures to reduce negative and maximise positive impacts. A health system based on community health will be better equipped to do this.

Comprehensive community health systems do not exist in high-income countries. Some countries including Australia, Canada, the United States and the United Kingdom have community health services that remain marginal to the bio-medically orientated mainstream. Community health systems are important because they focus on both the health system and the broader social factors that affect health.^1,2^ Community health services in high-income countries strive to provide comprehensive primary healthcare (PHC) but are hampered by the lack of a broad health system that also recognises the importance of action on the social determinants of health.^3^ The existence of parallel systems of PHC mirror international developments. In 1978 the philosophy and service model evident in community health systems was echoed in the World Health Organization’s (WHO’s) Alma Ata Declaration on PHC.^4^ This declaration, which has in part been revitalised in the WHO Astana Declaration,^5^ called for a new economic order, empowering democratic participation in health, and greater attention to social and environmental contexts that increased disease risks. The comprehensive vision of PHC was to be based on multi-disciplinary services, attuned to local need, and emphasising disease prevention and health promotion.^4^ The comprehensive Alma Ata model was very quickly labelled as too idealistic and a call for a more ‘selective’ PHC approach was published just one year later^6^ which envisioned a more ‘selective’ implementation as an ‘interim’ measure. The more selective approach promoted action on specific diseases rather than offering a holistic approach to the health of local communities and their population.

 Since the 1980s the attempts to establish community health systems which are modelled on the original Alma Ata vision have been largely unsuccessful. There really are no system-wide examples in high income countries where community health is regarded as the mainstream model. Most typically, community health services are regarded as models for disadvantaged areas, or focus on groups experiencing disadvantage such as Indigenous peoples and migrants and refugees.

The history of community health in Australia provides a case study by which to understand the barriers to the establishment of community health systems in high income countries. Two distinct strands of PHC provision developed in Australia from the 1970s onwards. The mainstream strand has been fee-for-service private general practice which since 1984 has been largely financed from Medicare (a national health insurance scheme) and, in some instances, additional out-of-pocket expenses. Alongside this system, community-driven health services evolved which are more concerned with accessibility, equity, action on the social determinants of health, community empowerment, and holistic and multi-disciplinary responses to health issues. The evolution of these services was supported by social movements that identified their need and fought to establish their own services or to encourage governments to support them. These proponents included Aboriginal health, trade unions, women’s health movements and locality-based community groups. The progressive Community Health Program (1972-1974) was “an innovative program designed to extend and reform the existing health system by encouraging a shift towards prevention, a focus on local communities and emphasis on PHC.”^7^ The national Community Health Program lasted only a few years and was not taken up by all states. Some states responded by expanding their own community health services, most notably New South Wales, South Australia and Victoria, albeit each with different models. However, the models shared a focus on multi-disciplinary services, community development, action on the social determinants of health, and local control and management.^8,9^ By 2020 in Australia, the comprehensive approach is best represented in Aboriginal community controlled services and 31 independently managed community health services in Victoria. Many community managed health services that developed through the 1980s and 1990s have been defunded or brought under direct public service control and now offer selective rather than comprehensive PHC.

 This paper will draw principally on two Australian research programs: one on community health services and one on regional PHC organisations. We used an action research approach. This enabled us to adapt to a fast changing environment which saw sweeping changes to the residual community system to the extent that it was mostly dismantled by the conclusion of the study. Our second study demonstrated the strong bias towards primary medical care in the regional PHC organisations. The aim of this paper is to examine the factors that account for the absence of strong community health systems in high income countries, using Australia as an example.

## Methods

 In this paper we interrogate data from two recent large Australian PHC studies led by the authors and funded by the Australian National Health and Medical Research Council: the Comprehensive PHC project, and the Regional PHC Organisation project.

The Comprehensive PHC project was a 5-year (2009-2014) action research project in partnership with 7 community health services. The characteristics of the comprehensive PHC partner sites are summarised in Table 1. We developed a program logic model with each service to portray how they implemented comprehensive PHC,^10^ and conducted methods at each service to understand how the model was implemented and what the perceived benefits were.^11-17^ The program logic was developed for each service (n = 7) with service staff participating in two workshops to develop and then approve the model.^10^ The generic version (Figure) drew on each program logic from the individual services. The model describes the mechanisms underpinning comprehensive PHC, the service qualities, the range of activities, the outcomes of these activities for individuals and communities and the population health outcomes. The logic models then informed the design of the subsequent research methods.

**Table 1:** Characteristics of the Case Study PHC Services in 2013

	**Budget (Per Annum, AUD)**	**Main Source of Funding**	**Approximate No. of Staff (FTE)**	**Examples of Disciplines Employed**
**Service A**	$0.5m^a^	State government	10 (8.1)	Social worker, speech pathologist, occupational therapist, dietitian
**Service B**	$1.3m^b^	State government	28 (15.7)	Nurse, doctor, podiatrist, social worker, PHC worker, speech pathologist, lifestyle advisor, dietitian
**Service C**	$1.6m	State government	25 (15.3)	Nurse, dietitian, speech pathologist, psychologist, occupational therapist, social worker
**Service D**	$0.6m^1^	State government	13 (12.8)	Aboriginal health worker, aboriginal PHC worker Aboriginal primary mental health support worker, youth workers
**Service E**	$1.7m	State government	21 (16.6)	Social worker, dietitian, psychologist, speech pathologist, nurse, occupational therapist, CHW
**ACCHS**	$20m	Federal government	310 (204.5)	Medical officer, psychologist, social worker, youth worker, midwife, nurse, Aboriginal health worker, pharmacist
**Sexual health NGO**	$5.8m	State + Federal government	68 (50.7)	Medical officer, nurse, counsellor, education coordinators, disability worker, aoriginal youth support worker

Abbreviations: AUD, Australian dollars; FTE, full time equivalent; ACCHS, Aboriginal community-controlled health service; NGO, non-governmental organization; PHC, primary healthcare; CHW, community health workers. ^a^ Approximate – budget hard to isolate due to restructures. ^b^ As of 2011, due to service withdrawing.

**Figure:**
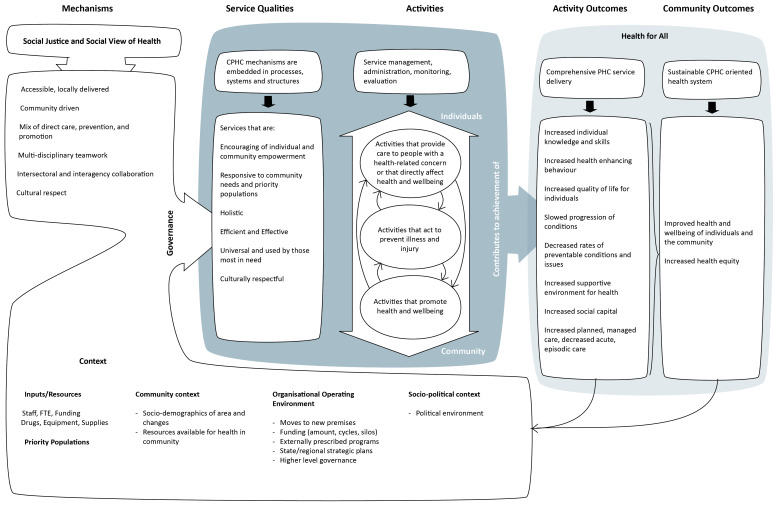
Southgate Model of Comprehensive Primary Healthcare (CPHC). Abbreviation: FTE, full time equivalent; PHC, primary healthcare.

The Regional PHC Organisation research was a 4 year (2014-2018) project that examined population health planning in Regional PHC organisations established by the Australian Federal government firstly as Medicare Locals (established in 2011), and then followed their transition to Primary Health Networks (in 2015). These organisations were designed to fill service gaps in PHC and plan services particularly to reduce demand on hospital services. We analysed publicly available documents from Medicare Locals and Primary Health Networks, surveyed and interviewed staff and board members, and Federal Department of Health staff, and conducted focus groups with Aboriginal and Torres Strait Islander, migrant, and mental health organization representatives.^3,18-21^ Key characteristics of the two forms of regional PHC organisations are shown in Table 2.

**Table 2:** Main Characteristics of Medicare Locals and Primary Health Networks in Australia

	**Medicare Locals**	**Primary Health Networks**
Time period	2011-2015	2015-current
Number	61	31
Governance	Each Medicare Local had a board. There was a national alliance body funded by the Federal government.	Each Primary Health Network has a board, clinical council, and community council. No peak organization.
Service delivery	Service delivery with some Medicare Locals commissioning.	Commissioning body only, no service delivery.
Key objectives	1. Improving the patient journey through developing integrated and coordinated services 2. Supporting the clinicians and service providers 3. Identifying and responding to local health needs 4. Facilitating the implementation of PHC initiatives and programs 5. Operating under a strong governance and effective management framework^22^	Seven stated priorities^23^: 1. Mental health 2. Aboriginal and Torres Strait Islander health 3. Population health 4. Health workforce 5. Digital health 6. Aged care 7. Alcohol and other drugs

Abbreviation: PHC, primary healthcare.

This paper draws on published work and re-examined data from both projects and applied the three Is (Interests, Institutions, and Ideas) theoretical lens^24^ to identify factors that discourage Australia from adopting a comprehensive community health system. The three Is approach uses institutional theory to understand how problems are defined, what policy options are selected, and how they are implemented. This framework was selected because it can identify ideas, agency and power as well as structures, and has been widely used in the health field.^25-27^ ‘Interests’ refers to actors’ (organisations or individuals) interests which may shape policy and practice, such as professional interests to safeguard their profession’s role.^20,23,26^ ‘Institutions’ refers to the written or unwritten rules and structures that govern how the health system is comprised and behaves, which is useful for identifying institutional drivers in the regulatory environment such as funding mechanisms and the mandates or goals of organisations.^3,19,26^ ‘Ideas’ are the philosophies and conceptualisations that underpin different approaches to health system organisation and service delivery, and can include neoliberalism, managerialism, and biomedical or social views of health.^3,19,26^

We reviewed the published work from both projects, and re-analysed the primary qualitative data using QSR Nvivo 12, to identify: (*a*) the main actors in the Australian PHC institutional field, (*b*) the “strife of interests”^3,28^ evident in the institutional field of PHC in Australia, and (*c*) the institutional forces and ideas that shaped the strengths and constraints on community health. Both authors contributed to the analysis, and draft findings were shared, debated and refined to improve rigour.

## Results

 We first consider the strengths of the community health system in Australia, and then their operating context to identify the barriers to implementing this model across the Australian system.

###  Strengths of the Community Health Model in Australia

 We documented a range of strengths of the previous ways of working of the state-managed community health services and persisting strengths at the aboriginal community-controlled health service (ACCHS) and sexual health non-governmental organisation (NGO). These are: accessibility and equity; community participation and responsiveness; multidisciplinary teams; and strategies beyond individual care.

*Accessibility and equity*. Community health services in Australia^7,29^ and globally^30^ have been explicitly underpinned by a philosophy of equity, local provision of services, and accessibility. Community health services were often established in areas of disadvantage to improve the health of these communities.^31^ We found this was the case for all comprehensive primary healthcare (CPHC) project services, and that all services put considerable effort into reaching those most in need.^14,15^ This included prioritisation of populations such as Aboriginal and Torres Strait Islander peoples, and people on a low income, provision of transport and creche services,^15^ community development activities such as community gardens that acted as a non-threatening entry point,^32^ and particularly at the ACCHS, outreach into the community, such as Aboriginal Health Workers who would go out to where homeless Aboriginal and Torres Strait Islander people lived. Some Medicare Locals did have an equity focus and engaged in some activities to improve accessibility and equity in PHC,^21,33^ though there is less evidence of their successors, Primary Health Networks considering equity in their role as commissioners of PHC services.^34^

*Community participation and responsiveness*. The ACCHS had full community control, with a board with elected community members, as well as other strategies to engage with community and gain community input into decision-making.^16^ While the state managed services had lost their community boards, they still pursued some community participation within the limited scope allowed by the state system.^16^ The sexual health NGO had advisory and reference groups, and Youth Action Teams that built capacity of young people, and provided avenues to contribute to design of education materials, and service decision-making.^16^

These participation and engagement strategies supported the services’ ability to be responsive to local needs, albeit within very narrow funding and mandate constraints on the state managed services. There were examples at state-managed and non-government services of needs identified in the community to which services were able to respond. One example at a state-managed service was identifying a local migrant population in poor quality housing, and establishing a tool library and living skills program in partnership with other community groups to encourage community interaction and capacity to repair their housing.^11^

Medicare Locals’ population health planning involved community engagement and consultation,^19^ and Primary Health Networks are mandated to have community advisory councils. However, interviewees reported difficulties in how best to incorporate community perspectives and the community advisory council members we interviewed reported misgivings about the extent of their influence. While some benefits were identified our study raised concerns about the extent of real voice or power community members had in Primary Health Networks’ decision-making which appeared to remain driven primarily by medical perspectives.

*Multidisciplinary teams*. One strength that did persist at all services was a focus on multi-disciplinarity (see Table 1). Staff reported generally much less hierarchical dynamics than would be typical in other health services such as hospitals, and enacted a range of strategies to provide holistic, coordinated care to clients, including joint appointments, case conferencing, and team planning for clients.^35^ This allowed, for example, a client with diabetes to see a dietitian, exercise physiologist, diabetes nurse educator, and podiatrist at the one service, to support many different aspects of their management of their diabetes. This provided a more whole-of-person approach to care than can be achieved in more selective primary medical care services. Medicare Locals extended on the previous divisions of general practice by having a more multidisciplinary focus in PHC planning, involving general practitioners (GPs), pharmacists, nurses, and other allied health in their remit and governance. Despite resultant GP dissatisfaction being one of the drivers of the replacement of Medicare Locals with Primary Health Networks,^36^ some of this multidisciplinary approach has continued with Primary Health Networks.

*Strategies beyond individual care*. Services engaged in a range of activities that went beyond individual appointments with clients. All services engaged in group work, such as health education and therapeutic groups, eg, information sessions for people with diabetes, and developmental and health promotion skills groups including cooking courses, supermarket tours, and community-oriented activities such as community lunches and cultural days.^13^

Services collaborated with other sectors to address social determinants of their community’s health.^11^ Examples included a learning centre for aboriginal community members to build job skills, the ACCHS advocating for alcohol supply measures, and participation in a domestic violence network.^11^ The services, particularly the two Aboriginal services, advocated for individuals for example by writing letters of support for court, welfare and housing.

These strengths were most amplified, and most persistent, in the ACCHS. It is consistent with the general philosophy underpinning ACCHS and points to the particular, enduring strengths of this model.^37^ ACCHS have sustained a comprehensive, holistic approach to community health since the early 1970s in Australia.^38^

While we found some evidence of Medicare Locals funding and engaging in activities that went beyond individual care, there was less evidence of this at Primary Health Networks.^3,33^ Primary Health Networks’ main focus has been on the commissioning of clinical services for individual care. However, the focus of both Medicare Locals and Primary Health Networks on population health planning approach goes beyond the provision of individual care and could be the basis of broader population health if they had support from the Federal Department of Health.

###  Barriers to a Community Health System

 We documented the move of the state managed services (Services A-E), and to a lesser extent, the sexual health NGO, away from a more comprehensive ethos, to a more selective, individual-focused and medical model of healthcare. We also found that Medicare Locals and Primary Health Networks have been dominated by the selective, medical model of PHC as the policy of the Federal government made this model a condition of funding.

Here we consider the barriers to the establishment of a comprehensive community health system in Australia. Drawing on both studies, we consider the barriers under the broad heading of ideas that shape healthcare provision, the interests that shape the system and the institutions which reflect the rules and structures in place (see Table 3).

**Table 3:** Ideas, Institutions and Interests Which Shape Current PHC Policy and Implementation in Australia and Potential Community Health System

**Mainstream PHC in Australia **	**Community Health System **
**Ideas**	
Medical model of care with focus on cure and some rehabilitation of individuals	Social perspective on health: Focus on care, prevention, promotion of whole community’s health
Neo-liberal ideas dominant in public discourse stressing market models and individualism	Public spending as a public good
**Interests**	
Private general practice privileged. Medical lobby groups have a strong voice in policy and very weak voice for community health	General practice as one part of multi-disciplinary team Health service user and citizen voices important and valued by policy-makers
**Institutions**	
Private provision from public funding: General practices operate as small businesses. Cost cutting as explicit aim and floating of privatisation as option. Increasing user fees. Commissioning services from NGOs and private services	Public sector funding to ensure equity of access and to contribute to equity of outcome
Professional decision-making most valued	Structured avenues for community voice including community control through boards of management

Abbreviations: PHC, primary healthcare; NGOs, non-governmental organizations.

###  Clash of Ideas

 Both studies demonstrated the persistence of the dominance of a medical model of health in health policy and public services. Community health services had been the one place in the health system where social health ideas were able to flourish. Some of our interviewees working in services and in policy positions described how sophisticated understanding of the influence of social factors on the health of individuals and communities was greatest among those working in community health services. The work of the services was grounded in an understanding that disease and poor health had their roots in the social and economic conditions in which people lived. Thus the community health staff spoke of the ways in which they would take these social determinants into account during service delivery. Examples are working with the housing department or homelessness service to find accommodation for a homeless client or running a successful peer-to-peer food program that focused on skills and empowerment rather than one-way messages. However, group and community development work were completely curtailed in the state services.

While most community health services have been nominally universal, in practice their marginal status and insufficient funding has meant community health services have been better typified as residual – largely seeing prioritized subgroups of the population such as Aboriginal and Torres Strait Islander people, or people on a low income, with other typically more advantaged groups in the population using mainstream primary care services instead.^12,31^ At the same time, constraints on funding and mandate have made services hard to reach for many people experiencing disadvantage or marginalisation.^14^ Managerialist approaches to community health services constrained services’ accessibility, through emphasizing individual throughput, and reducing supportive services such as transport and creche, community outreach, and group and community work that supported access.^12,14^ The regional PHC organisations Medicare Locals were expected to pick up some of this focus,^39^ but their actions have been limited.^33^ By contrast the ACCHS expanded their transport service as they saw accessibility as vital to the effectiveness of the health services they offer.^13^

Advocacy on social determinants was curtailed in the state-funded services. The ACCHS was able to continue because of the strong social health framework adopted by the services’ management team and board.^37^ Medicare Locals had some capacity to respond to social determinants and one was specifically funded to mount such a response. By contrast our respondents were very clear that PHNs contracts did not include social determinants. One noted:

*“The sad fact is that social determinants of health are not part of our PHN KPIs. They are still very much our priority but they sit over and above what the government expects us to be able to deliver on so that makes it more difficult to devote the resources to it when that’s not the government’s priority of action*”^3^ (p. 4).

The second clash of ideas concerned the ways the dominant neo-liberal ethos in both state and federal governments discouraged strong community health systems. All the community health services in our study were publicly funded. The ACCHS and the sexual health service operated as incorporated NGOs but received nearly all their funding from state and federal governments. The others were directly funded and managed within the state health service. The health system in Australia has deep roots in bio-medicine.^28,40^ This means that community health services hold a marginal position within the state health system with little power compared to the acute care services. This powerless position meant they found it hard to defend the sector against cuts. Indeed, when these services were transformed from community health services to intermediate care centres providing care to those with chronic disease there were no strong health system voices in favour of community health. By contrast when changes affect medical practitioners there are very loud voices from the Australian Medical Association. The only co-ordinating body – the Primary Health Networks – were funded by the Federal government with a brief to focus on private general practice and so do not offer support for more comprehensive community health services.

###  Interests: Professionals and Communities 

 Both of our studies found that professional interests often overrode community interests. This was shown by the ways in which the interests of GPs were privileged. They are the only professional group to receive most of their funding through the universal health insurance scheme Medicare. Most GPs in Australia are in private fee-for-service practice. The opposition of organised medicine to any changes to this arrangement has seen some community health services established without any GPs. This was the case in three out of the seven in our study. The Primary Health Networks had an explicit aim of refocusing on private general practices at the expense of a more multidisciplinary approach.

The great promise of community health systems is that they would prioritise community interests in the health system. To do this they require effective means of community decision-making. The most robust model of putting that principle in to action was that of the ACCHS. The state government funded community health services had lost their boards of management in the early 2000s and afterwards adopted more bureaucratic practices which saw a gradual move away from the community orientation to a professional one. An example of what this change meant in practice was that staff were told to see a quota of clients and were not allowed to continue community engagement or group work. A further example was the reduction in support for the community lunches and cessation of annual health camps at one service run for aboriginal people, intended as a mechanism to engage people whose lives were often chaotic and unsettled. These events served as a mechanism to engage aboriginal people and build their trust in the health services. Such engagement is rooted in a social model of health and this approach was crowded out by an exclusive medical model. A prioritisation of a more professional orientation was evident at the Primary Health Networks: in our analysis of their boards, all boards had multiple people with a medical background (very often including the Chair), but only 5 of 29 had a community representative. Clinical advisory committees were mandated for Primary Health Networks alongside their community advisory councils but the latter committees did not hold much power. Our consultations with community groups working with migrants and refugees^21^ found that Medicare Locals were not very active in providing services for this group and this reduced over time.

###  Institutional Drivers: Management Reforms Driven by Neoliberalism

Community health services were constrained by the imposition of managerialism and austerity driven policies that came as part of the neo-liberal policy frame evident in the state Department of Health.^12^ For the community health services this was experienced as a period of considerable uncertainty with a high turnover of management staff and budget cuts with consequent poor morale.^41^ They also experienced an increasing focus on short term measurable outputs of their establishing the throughput of clients as the key performance indicators rather than those associated with the hallmarks of community health such as collaborations with other sectors, community empowerment and group activities.^17^ The logic of this system was based on the needs of acute care rather than engaged community work.

Primary Health Networks’ commissioned services were overwhelmingly for individual services rather than community activities. The Australian Government opened the way for the private health insurance industry in the governance of the Regional PHC Organisations. Mixed views were reported on this involvement. Some expressed cautious optimism and that it would make sense for them to be involved in primary care, but others reflected the view that you had to ask “if it’s a private organisation whether they’ve got other interests at heart” and that “it’s just hard to find a point of confluence.”^3^ The pressure to involve private partners however, was a clear indication that the policies were not in accord with the establishment of a community health system.

## Discussion

This study used secondary data analysis to build on our existing research findings to identify drivers of the failure of the community health system to thrive in Australia. The three Is framework^42^ served as a valuable analytical theory to synthesise prior research and illuminate overarching drivers. The limitation is that we did not undertake primary research to directly answer our research question but drew on our previous studies which examined the strengths of the comprehensive PHC model.

Our two studies of elements of the Australian healthcare system have enabled us to examine the factors that discourage an effective community health system being developed and leads to lessons that are likely to be applicable to other high-income countries. While our study mapped a move away a community health system we were still able to document the benefits of a more comprehensive system. In the discussion we focus on elements that our research suggests will encourage the adoption of community health systems in high income countries: a supportive government policy environment, and health sector policy support for community participation, social health, and a multidisciplinary service model. Table 4 displays these elements and lists how they would need to be supported in terms of ideas, interest and institutional factors.

**Table 4:** What Elements Supported by Which Ideas, Interests and Institutions Are Required to Establish a Community Health System in a High-Income Country?

	**Ideas**	**Interests**	**Institutions **
Overarching government policy environment	Economic theories that focus on people’s rather than market needs Acceptance of government responsibility for community well-being	People and community interests dominant Professional economic interests are not allowed to dominate	Popular movement driven civil society has strong influence which is structurally part of governmental processes Overall government goal to improve well-being with funding to support and organisational commitment
Health Sector policies and practices	Social model of health accepted and promoted through policies and practices Value of comprehensive PHC is promoted as essential for whole population not as residual service for disadvantaged	Community interests promoted above medical and other health professional Not high tech medicine for some but equity in access and outcome for all	Community governance instituted and supported building on existing successful models such as Australian Aboriginal Community Controlled Health Services
Multi-disciplinary service models	Multi-disciplinary models better meet the needs of patients and communities Therapeutic ideas and community development are equally valued	No one profession is dominant	Structures are built to encourage effective multi-disciplinary working Reward structures are equal for community work and promotion activities as for individual therapeutic work

Abbreviation: PHC, primary healthcare.

###  Overarching Government Policy Environments 

The reforms in South Australia that undermined the community health system are mirrored in Australian and international literature.^43-47^ The reforms sought to reduce public spending including by reducing public sector employment and increased the focus on easily measurable throughputs. These neo-liberal reforms did not encourage long term disease prevention and emphasised efficiency measured through short term outputs rather than longer term health outcomes. Strong community health systems will best be supported in policy environments which encourage investment in long term community development and establish goals which are broad and whose success is judged by the vitality and sustainability of community initiatives.^48^ Davis^49^ unpacks the recent adherence to neo-liberalism in Australia and notes that it was always about more than economic policy and was part of “a broader conservative movement generally opposed to such things as planned forms of wealth redistribution, social collectivism and communism, special rights for minorities and women, ‘elites’ and ‘big government’” (p. 32). This neo-liberalism has spread through the world and its existence is a threat to the collectivist and redistributive aims of community health systems. Community health systems require governments that accept responsibility for their communities’ well-being and adopt economic models which aim to achieve this. There is much current thinking about such economic models which have been reviewed by Baum.^50^ Korten^51^ questions the need of continual economic growth and the need to reduce the power of trans-national corporations and ensure that taxation is sufficient to fund the mechanisms needed to ensure community well-being.

Instituting a community health system is likely to be the result of civil society demands and advocacy. Lefkowitz^52^ describes the history of community health centers in the United States and describes how they emerged as part of the civil rights movement and provided not only primary and preventive services but also social services, economic development and empowerment. The United States, Canadian and Australian community health centres have always remained marginal to the mainstream but at periods in their history have benefited from more supportive government environments and social movements.

###  Health Sector Policies and Practices 

Health policies in Australia have become increasingly neo-liberal and less supportive of community health. When the Community Health Program was established in 1973, policies which supported community management and the notion of health professionals being advocates for their community were evident. In the 1980s the inspiration of the WHO Alma Ata Declaration was seen in public policy through national programs encouraging the implementation of Health for All^29^ and the expansion of community health in the states of Victoria and South Australia.^7^ While the community health system remained marginal to the mainstream it was supported by health bureaucracies which drafted policies such as the South Australian Social Health Policy^53^ and a District Health Council program in Victoria.^54^ These indicate a health system that supported the defining features of community health systems including community control and a social health model. Community management became increasingly out of fashion from the 1990s. The exception in Australia has been ACCHS. These have been established as NGOs but receive the overwhelming amount of their funding from government. They have a strong peak body – the National Aboriginal Community Controlled Organisation^55^– which is a powerful voice in national health policy. In local communities, boards are respected and ensure services keep contact with community needs.^37^ The links to local communities and their presence in management has also been credited with the continuance of the US community health services despite a very unsupportive private health system.^52^ These experiences show the importance of a strong social movement to support a community-controlled system. But they also demonstrate that a social movement rooted largely within disempowered and poor communities would need to gain political will in order to make the community health system mainstream.

Finally, community health systems are unlikely to flourish in a health system based on private, for profit care as the drivers for such a system are personal care and the importance of making a profit for the provider or health insurance company. A social health view is unlikely in a privatised health system because the values of solidarity, communal benefits and social justice are not emphasised in a for-profit system. In such a system community health will be a residual service targeted at people living in disadvantage as the community centers in the United States are. Most health budgets in high income countries are spent in hospitals on interventions producing very marginal population health gains and often represent futile care. The Lancet^56^ has supported a movement for Right Care which examined the extent of overuse and underuse of medical services. It found some services that are more likely to cause harm than good and that in other cases appropriate care is not received. Redirection of funds from expensive medical technology to the community health sector will be required and will be difficult to achieve given the existence of a medical and pharmaceutical industry making its profits from this aspect of medicine.^57^

###  Multidisciplinary Service Model 

Community health systems rest on multidisciplinary teams which include but are not necessarily led by doctors. Yet many health systems are dominated by the discipline of medicine which has deep institutional roots in all high-income countries. The power of the medical profession is so strong that it is rarely questioned and is generally taken for granted.^58^ While medicine offers many benefits and it is an essential part of a community health system, its individualised practice is not sufficient to establish a community health system that responds to the illness of individuals as well as the health and well-being of the population. A comprehensive service relies on a range of curative services including general medical practice, speech pathology, physiotherapy, occupational therapy, psychologists, social workers, nutritionists, and community nurses. Just as importantly the service requires workers who work with people to assist them gaining greater control over their lives through running groups or developing community projects which focus on areas of community improvement such as advocating for improved public transport, less air pollution from local industry, a clean up of a local river or the establishment of a children’s playground. Community health services can organise community engagement events such as the community lunches provided by the Aboriginal service in our study. Thus the skills of health professionals need to go beyond those of individual care to encompassing these broader community development skills.^59^

Our study found limited use of community health workers (CHWs) outside of Aboriginal Health Workers. CHWs are most typically associated with low- and middle-income countries’ health systems. Yet their value in high income countries is also likely to be great despite the institutional reluctance to appointing such workers, shown by the opposition of health professional groups to task shifting to CHWs.^60^ A small Australian study^61^ concluded that CHWs (including aboriginal health workers) serve a range of functions in Australian PHC with their main function being facilitating access to services and information and that they enhance service access for communities facing disadvantage. A systematic review on CHWs^62^ found 29 studies on CHWs in high income countries. The review found that effective integration of CHW programs into health systems can bolster program sustainability and credibility and recommended the model as being relevant to high income countries. There is some evidence that high income countries are studying the lessons for their own system from the use of CHW in low- and middle-income countries. For example, Wales has examined the Brazilian model of CHW to determine what is applicable to their setting.^63^ Consequently, we conclude that a community health system in high income countries would be strengthened by the inclusion of CHWs as part of their multidisciplinary teams.

 This study used secondary data analysis to build on existing research findings to identify drivers of the failure of the community health system to thrive in Australia. The three Is framework served as a valuable analytical theory to synthesise prior research and illuminate overarching drivers. The limitation is that we did not undertake primary research to directly answer our research question.

## Conclusion

 This paper has considered the questions of why community health systems don’t flourish in high income countries. We identified the barriers as the neo-liberal orientation of policy, the focus of health systems on treatment rather than prevention and promotion, the dominance of the medical profession in PHC, the lack of policy encouragement and initial training for interprofessional team work and on the social determinants of health. Instituting a community health system in high income countries will require systematic change in each of these areas and strong political and institutional will to make this happen.

## Acknowledgements

 We thank our co-investigators on the two NHMRC grants from which we conducted analysis for this paper.

## Ethical issues

 Ethics approvals were received from the Flinders University Social and Behavioural Research Ethics Committee, the Southern Adelaide Clinical Human Research Ethics Committee, and the necessary health sector ethics committees.

## Competing interests

 Authors declare that they have no competing interests.

## Authors’ contributions

 FB led funding acquisition, research design, data analysis, and write up of the manuscript, and contributed to data collection. TF contributed to funding acquisition (for grant 1064194), research design, data collection, data analysis, and write up of the manuscript.

## Funding

 National Health and Medical Research Council [grant numbers: 535041, 1064194].
